# Biochemical and Molecular Characterization of a Flavonoid 3-*O*-glycosyltransferase Responsible for Anthocyanins and Flavonols Biosynthesis in *Freesia hybrida*

**DOI:** 10.3389/fpls.2016.00410

**Published:** 2016-03-31

**Authors:** Wei Sun, Lingjie Liang, Xiangyu Meng, Yueqing Li, Fengzhan Gao, Xingxue Liu, Shucai Wang, Xiang Gao, Li Wang

**Affiliations:** ^1^Institute of Genetics and Cytology, Northeast Normal UniversityChangchun, China; ^2^College of Life Science, Guizhou Normal UniversityGuiyang, China; ^3^Key Laboratory of Molecular Epigenetics of MOE, Northeast Normal UniversityChangchun, China

**Keywords:** *Freesia hybrida*, flavonoid, flavonoid 3-*O*-glycosyltransferase, substrate specificity, regiospecificity

## Abstract

The glycosylation of flavonoids increases their solubility and stability in plants. Flowers accumulate anthocyanidin and flavonol glycosides which are synthesized by UDP-sugar flavonoid glycosyltransferases (UFGTs). In our previous study, a cDNA clone (*Fh3GT1*) encoding UFGT was isolated from *Freesia hybrida*, which was preliminarily proved to be invovled in cyanidin 3-*O*-glucoside biosynthesis. Here, a variety of anthocyanin and flavonol glycosides were detected in flowers and other tissues of *F. hybrida*, implying the versatile roles of *Fh3GT1* in flavonoids biosynthesis. To further unravel its multi-functional roles, integrative analysis between gene expression and metabolites was investigated. The results showed expression of *Fh3GT1* was positively related to the accumulation of anthocyanins and flavonol glycosides, suggesting its potential roles in the biosynthesis of both flavonoid glycosides. Subsequently, biochemical analysis results revealed that a broad range of flavonoid substrates including flavonoid not naturally occurred in *F. hybrida* could be recognized by the recombinant Fh3GT1. Both UDP-glucose and UDP-galactose could be used as sugar donors by recombinant Fh3GT1, although UDP-galactose was transferred with relatively low activity. Furthermore, regiospecificity analysis demonstrated that Fh3GT1 was able to glycosylate delphinidin at the 3-, 4-′, and 7- positions in a sugar-dependent manner. And the introduction of *Fh3GT1* into *Arabidopsis UGT78D2* mutant successfully restored the anthocyanins and flavonols phenotypes caused by lost-of-function of the *3GT*, indicating that *Fh3GT1* functions as a flavonoid 3-*O*-glucosyltransferase *in vivo*. In summary, these results demonstrate that Fh3GT1 is a flavonoid 3-*O*-glycosyltransferase using UDP-glucose as the preferred sugar donor and may involve in flavonoid glycosylation in *F. hybrida.*

## Introduction

Flavonoids, the important polyphenolic secondary metabolites, are widely distributed in plant species and possess diverse biological functions including pollination, pigmentation, auxin transport inhibition, UV light protection and other defense mechanism (Brenda, 2001; Koes et al., 2005; Charles et al., 2010). In plants, some biological processes, such as cell-to-cell communication, signal transduction, and transcriptional regulation are also affected by flavonoids (Almudena et al., 2012). Flavonoids consist mainly of anthocyanins, phlobaphene pigments, and proanthocyanidins, as well as the flavonols, flavanones, and isoflavonoids (Grotewold, 2005; Taylor and Grotewold, 2005). Among them, anthocyanins are broadly existed in flowering plants to provide flowers and fruits with the red, purple, and blue pigmentation for attracting pollinators or seed dispersers. Flavonols, colorless co-pigments, affect the brightness and brilliance of colors and have vital roles in pollen germination (Ferreyra et al., 2012; Zhao and Tao, 2015). In addition, anthocyanins and flavonols also play an important role in human health and have potential medicinal uses, as the intake of them can protect against cardiovascular disease, cancer, and many other diseases (Petroni et al., 2014; Santos-Buelga et al., 2014).

**FIGURE 1:**
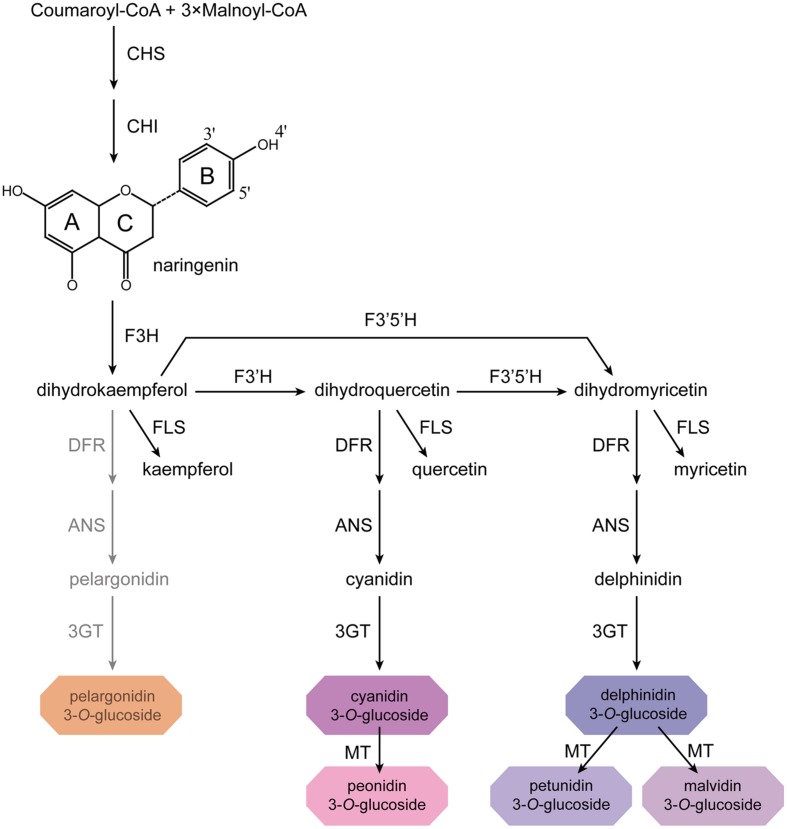
**Proposed flavonoid pathway in *Freesia hybrida* flower.** Hoary areas indicate the biosynthesis of pelargonidin-based pigments is blocked.

The biosynthesis of anthocyanins is derived from the flavonoid branch of the phenylpropanoid metabolic pathway and has been well studied in different plants including *Zea mays*, *Petunia hybrida*, *Arabidopsis thaliana*, and *Antirrhinum majus* (Gonzalez et al., 2008; Petroni and Tonelli, 2011; Wang et al., 2013). The anthocyanin biosynthetic pathway starts with the condensation of 4-coumaroyl-CoA with three molecules of malonyl-CoA, leading to the formation of naringenin chalcone by CHS (**Figure 1**). Next, the naringenin chalcone is isomerized to naringenin through the action of CHI. After hydroxylation at the three position of naringenin, dihydroflavonols are formed and will be further catalyzed to yield leucoanthocyanidins by DFR. Furthermore, FLS can also use dihydroflavonols as substrates to produce flavonols. Subsequently, the action of ANS generates the anthocyanidins which are then converted to glucosylated anthocyanins by UDP-glucose: 3GT (Tanaka et al., 2008). Recent studies have demonstrated that nearly all the enzymes involved in anthocyanidin biosynthesis have been isolated and functionally characterized (Quideau, 2006; Almudena et al., 2012), but the sequential modification of anthocyanidins metabolism, such as glycosylation, methylation and acylation, remains relatively unexplored.

Many anthocyanins as well as other flavonoids are known to exist in glycosylated form in plants. During the biosynthesis of these glycosides, glycosylation is often the final step and serves a variety of roles in plant metabolism. For example, glycosylation can enhance the stability and solubility of the acceptor molecule and affect their subcellular localization (Thomas and Patrik, 2000; Wang, 2009). Meanwhile, it also regulates cellular homeostasis and plant growth, or may involve in the detoxification of exogenous toxins (Lim and Bowles, 2004; Brazier and Edwards, 2005). The enzymes that catalyze the formation of glycoside are known as uridine diphosphate (UDP): flavonoid glycosyltransferases (UFGTs), which transfer UDP-activated sugar moieties to low molecular-weight acceptor aglycone. UFGTs can be identified by the presence of PSPG (Plant Secondary Product Glycosyltransferase) box, a 44-amino acid conserved motif that is involved in binding substrates to the UDP moiety of the sugar donor (Joe et al., 2001; Gachon et al., 2005; Offen et al., 2006). Several different UDP-sugars have been reported to be donors for UFGTs including UDP-glucose, UDP-galactose, UDP-rhamnose, and UDP-xylose. Crystal structures of UFGTs and mutation analyses have demonstrated that sugar selectivity is relatively determined by the last residue in the PSPG motif (Wang, 2009). Moreover, these enzymes exhibit broad substrate specificity but exert strict regioselectivity in many cases (Thomas and Patrik, 2000). For example, 3GTs from petunia and *Arabidopsis* transfer sugar donors to only 3-position of both anthocyanidins and flavonols (Mami et al., 2002; Takayuki et al., 2005). To date, genes encoding UFGTs have been cloned and identified in a number of plant species. According to the specificity for substrates, they can be divided into two groups. The first group is responsible for attaching a sugar to flavonoids, including flavonoid 3-*O*-glycosyltransferase (Ford et al., 1998; Mami et al., 2002; Zhao et al., 2012) and flavonoid 5, 3-*O*-glucosyltransferase (Jun et al., 2005). The second group catalyzes the glycosyl transfer toward flavonoid glycoside leading to the formation of diglycoside, such as flavonoid 3-*O*-glucoside 2”-*O*-glucosyltransferase (Morita et al., 2005). Among these glycosyltransferases, flavonoid 3-*O*-glycosyltransferases are best characterized, because of their essential roles in flavonoids biosynthesis (Jun et al., 1998; Griesser et al., 2008; Hu et al., 2011).

Anthocyanin and flavonol accumulation has significant effects on flower quality, and many *3GT* genes involved in the accumulation of anthocyanins and flavonols have been identified in dicotyledonous flower plant species, such as *Antirrhinum majus* (Martin et al., 1991), *Gentiana triflora* (Yoshikazu et al., 1996), *Senecio × hybridus* (Taylor and Grotewold, 2005), and petunia (Mami et al., 2002). However, only few studies have been reported on the characterization of *3GT* genes in monocotyledonous ornamental plants. Until now, only two *3GT* genes from monocotyledonous flower plants were confirmed to participate in the biosynthesis of anthocyanin (Yoshihara et al., 2005; Chen et al., 2011).

*Freesia hybrida* is one kind of monocotyledonous ornamental species that distributes widely in the world and belongs to the Iridaceae. Because of its diverse flower color, it could be chosen as an ideal material to study the biosynthesis of flavonoids. Moreover, functional characterization of *3GT* gene from *F. hybrida* may also contribute to the study of evolution of the *UFGT* gene family considering that very few of them have been identified in monocotyledonous ornamental plant. In our previous study, Fh3GT1 was found to be responsible for cyanidin 3-*O*-glucoside biosynthesis (Sui et al., 2011). However, its roles in the biosynthesis of other anthocyanins and flavonols are still unknown. To further elucidate the versatile functionality of Fh3GT1 systematically, we firstly performed the correlation analysis between the flavonoid glycoside products accumulation and the expression profiles of *Fh3GT1* in flowers at different pigmentation stages and in different plant tissues. Meanwhile, the biosynthetic pathway of anthocyanin in Fressia was also proposed on the basis of metabolites. In addition, substrate specificity and kinetic analyses were conducted to identify the activity of Fh3GT1 against various flavonoids. Furthermore, in order to confirm its function *in vivo*, *Fh3GT1* was transformed into the *Arabidopsis UGT78D2* mutant to restore the biosynthesis of anthocyanins and flavonols. In consequence, these results not only provide new insight to plant glycosyltransferase structure-function analysis but also useful for manipulating flavonoid biosynthesis in *F. hybrida* as well as in other monocotyledonous ornamental plants.

## Materials and Methods

### Plant Materials

*Arabidopsis* lines used in this study were in the Columbia ecotype background. Wild-type, T-DNA insertion line (*UGT78D2*), and transgenic seedlings of *Arabidopsis* (*Arabidopsis thaliana*) were grown on half-strength MS medium under a controlled condition (16 h of light/8 h of dark cycle at 22°C). For reverse transcription PCR (RT-PCR) and flavonoid analysis, whole seedlings were collected at 7 days after germination and stored at –80°C. Tissues and flowers at different developmental stages from *F. hybrida* were frozen in liquid nitrogen and kept at –80°C.

### Chemicals

UDP-glucose, UDP-galactose, quercetin 3-*O*-glucoside, delphinidin 3-*O*-galactoside, cyanidin 3-*O*-galactoside, delphinidin, cyanidin and malvidin were purchased from Sigma–Aldrich (USA), petunidin and peonidin from Tokiwa Phytochemical (Japan), pelargonidin, delphinidin 3-*O*-glucoside, cyanidin 3-*O*-glucoside, malvidin 3-*O*-glucoside, pelargonidin 3-*O*-glucoside, petunidin 3-*O*-glucoside and peonidin 3-*O*-glucoside from Phytolab (Germany), quercetin, kaempferol from ChromaDex (USA), and 3-*O*-glucoside of kaempferol from Extrasynthese (France).

### Subcellular Localization of Fh3GT1

GFP coding region without stop codon was amplified using primers (GFPF1 and GFPR1) that added *Xba*I sites to both the 5′end and 3′end of GFP. The fragments were then inserted into the *Xba*I-digested pBI121-*Fh3GT1* (constructed as below). The full length coding region of GFP was also amplified using GFPF2 and GFPR2 (**Supplementary Table S1**) then subcloned into the pBI121 empty vector to serve as a control for localization experiments. Sequence integrity of the vectors was confirmed by sequencing. The two constructs were transformed to *Agrobacterium tumefaciens* strain GV3101 using a freeze-thaw method and then introduced into *Arabidopsis* plants using the floral dip method (Clough and Bent, 1998). Transformants were selected on 1/2 MS medium containing 50 mg L^-1^ kanamycin. For observation, a small piece of leaf tissue of T2 plants was cut out and mounted in deionized water followed by confocal microscopy.

### HPLC Analysis of Anthocyanins and Flavonols Extracted from *Freesia hybrida*

For the analysis of anthocyanins and flavonols in flowers from *F. hybrida*, 0.3 g finely ground tissue was extracted with H_2_O:MeOH:HCl (75/24/1v/v/v) at 4°C for 12 h in the dark and subsequently centrifuged at 12,000 rpm for 10 min. After centrifugation, the supernatant was collected, filtered, and used for HPLC analysis. Chromatographic analysis was carried out on a Shimadzu HPLC system equipped with an autosampler with a 20 μl loop, a LC-6AD HPLC Pump and an ACCHROM XUnion C18 column (250 mm × 4.6 mm, 5 μm). The column was eluted with solvent systems A (5% formic acid in H_2_O) and B (methanol) under the following conditions: 0-10 min, 14–17% B; 10–35 min, 17–23% B; 35–60min, 23–47% B; 60–67 min, 47–14% B; 67–70 min, 14% B with a flow rate of 1 ml min^-1^. Detection was monitored at 520 and 360 nm for anthocyanins and flavonols, respectively, and the photodiode array spectra were recorded from 200 to 800 nm.

High performance liquid chromatography-electrospray ionization (ESI)-tandem mass spectrometry (MS) analysis used an API2000 mass spectrometer (AB Sciex) and a SPD-20AV UV/VIS Dectector (Shimadzu, Kyoto, Japan). This mass spectrometer was equipped with an ESI source. Ion Trap source parameters in positive mode were as follows: ESI source voltage, 4.5 kV; gas (N_2_) temperature, 450°C; declustering potential, +80 V; entrance potential, 10 V; and scan range, m/z 100–1000 units. Metabolites were identified by their retention times, mass spectra, and product ion spectra in comparison with the data of authentic standards. Quantitative analysis was done using the external standard curve calibration of delphinidin 3-*O*-glucoside and quercetin 3-*O*-glucoside standards (Fanali et al., 2011). The calibration curves used were linear (*R*^2^> 0.99) in the concentration ranges investigated. All the measurements were performed three times independently with three biological replicates.

### Expression Analysis

For real-time qPCR, total RNA was isolated from petals, stamens, pistils, calyxes, toruses, scapes, leaves, roots and flowers at different stages of development using RNAiso Plus (TaKaRa). Then total RNA extracted from the above samples was treated with RNase-free DNase I (TaKaRa) and reverse transcribed using M-MLV reverse transcriptase (Promega). Gene-specific primers (PF1/PR1) were designed using Primer Premier 5 and summarized in the Supporting Information (**Supplementary Table S1**). To investigate the gene expression profiles, real-time qPCR was carried out with ABI StepOne Plus Real-Time PCR System (USA) and SYBR Master Mix (TOYOBO). Thermal cycling conditions were 95 °C for 60 s, then 40 cycles of 95°C for 5 s and 60°C for 60 s, followed by a melting temperature cycle, with constant fluorescence data acquisition from 60 to 95 °C. As an internal control, the *18s rRNA* gene was used. To ensure a single amplicon had been generated, the real-time qPCR products were confirmed by agarose gel electrophoresis and DNA sequencing. Data of the gene expression was analyzed with the 2^-ΔΔCT^method (Livak and Schmittgen, 2001), and each data point represents the average of three independent experiments.

### Heterologous Expression of Fh3GT1 in *E. coli*

To express *Fh3GT1* in *E. coli*, the full length cDNA of *Fh3GT1* was amplified by PCR using *Premix Taq*^TM^ (TaKaRa) with the primers PF2 and PR2 (**Supplementary Table S1**). The PCR product was sub-cloned into the pET-28a (+) His-fusion protein expression vector, and clone authenticity was confirmed by sequencing. The constructed vector was transformed into *E. coli* BL-21 (DE3) cells for recombinant protein expression. Then, the transformants were pre-cultured at 37°C for 12–14 h in Luria Broth (LB) media containing 100 mg L^-1^ kanamycin. Two milliliter of the preculture was transferred to the fresh LB media (400 ml) containing the antibiotics and grown at 37°C until an *A*_600_ of 0.6 was reached. After the addition of 40 μl of 1 M isopropyl-β-*d*-thiogalactopyranoside (IPTG), the induced culture was further incubated at 16°C for 50 h. The cells were harvested by centrifugation, resuspended in 20 ml of phosphate-buffered saline (PBS, pH 7.4), and disrupted by sonication. After centrifugation at 12,000 rpm for 20 min, the supernatant was applied to a column containing 3 ml Ni Sepharose (GE Healthcare) that had been equilibrated with PBS. Bound protein was eluted from the column using 100 mM imidazole in PBS. The purified proteins were collected and analyzed on SDS-PAGE. Expressed proteins in the gels were stained with Coomassie brilliant blue R-250 for visualization.

### Recombinant Enzyme Assays

The standard reaction mixture for Fh3GT1 enzyme assay consisted of 100 mM potassium phosphate buffer (pH 8.0), 100 μM flavonoid substrates (except for *K*_m_ determination), 10 mM UDP-glucose and 30 μl enzyme extract (20–30 μg protein) in a total volume of 200 μl. The glucosyltransferase activity assays were performed at 30°C for 5 min and terminated by adding 50 μl HCl (5%). Reactions with protein extract obtained from BL-21 (DE3) cells that transformed with an empty pET-28a (+) vector were performed as controls. Subsequently, samples were centrifuged at 12,000 rpm for 5 min to collect the supernatant. After filtration through a 0.22 μm membrane filter, reaction products were analyzed by HPLC. Glucosylation products were determined by comparison of integrated peak areas of the glucoside to the corresponding standard curve which was verified to be linear over the range studied.

### Enzyme Specificity

Substrate specificity of Fh3GT1 was also examined against eight potential substrates including cyanidin, delphinidin, pelargonidin, petunidin, peonidin, malvidin kaempferol, and quercetin using 10 mM UDP-glucose as a donor substrate. In addition, 10 mM UDP-galactose was also tested using cyanidin and delphinidin acceptors. Enzyme assays were performed as described above with 100 μM of acceptor substrate and stopped at 5 min.

### Enzyme Kinetics

To determine apparent *K*_m_ values, the concentration of peonidin, delphinidin, and quercetin was varied from 12.5 to 275 μM, and 10 mM UDP-glucose was used as donor substrate. Enzyme assays were conducted as described above with 8 μg of the purified enzyme but reactions were stopped at 2 min where the reaction velocity is liner. K_m_ and V_max_ values were obtained from Lineweaver-Burk plots of initial rate data.

### Vector Construction and *Arabidopsis* Transformation

A pair of primer, PF3 and PR3 (**Supplementary Table S1**), was designed to amplify the full length coding regions of *Fh3GT1* gene, using cDNA from red flowers of *F. hybrida* as templates. *Fh3GT1* was cloned into *Xba*I/*BamH*I-digested pBI121 vector harboring the CaMV 35S constitutive promoter and confirmed by sequencing. Genetic transformation was performed as decribed above. T1 seeds were selected on 1/2 MS medium containing 50 mg L^-1^ kanamycin then transferred to soil to set T2 seeds. After 1 week of culture on anthocyanin gene induction media (Kovinich et al., 2010), three independent transgenic lines which accumulated visible levels of anthocyanins were subjected to further analysis. To confirm the expression of *Fh3GT1* in the transformed mutants, total RNA was isolated from T2 transgenic seedlings and used for RT-PCR. And the *Arabidopsis actin* gene was used as a control (Penninckx et al., 1996).

### Metabolite Analysis of T2 Transgenic Seedlings

Hundred microgram of 1-week-old *Arabidopsis* seedlings cultured on anthocyanin gene induction media (above) were ground in liquid nitrogen and submerged in 1 mL extraction solution at 4°C for 12 h. Extracts were centrifuged for 10 min at 12,000 rpm then filtered through a 0.22 μm filter, and 20 μl aliquots was analyzed by HPLC as described above. The content of anthocyanin and flavonol was determined by external standard curve calibration of cyanidin 3-*O*-glucoside and quercetin 3-*O*-glucoside standard, respectively (Fanali et al., 2011). Data were represented as mean values from three independent experiments with three replicates each. Statistical significances of the differences were determined using Student’s *t-*test. Differences between *Arabidopsis* lines were considered significant when *P* < 0.01.

## Results

### Analysis of Fh3GT1

Analysis of Fh3GT1 sequence for the N-terminal targeting signal or C-terminal membrane anchor signal using the SignalP and TargetP Web-based programs predicted a signal peptide localized between amino acids 20 and 21. Prediction of subcellular location with PSORT II^1^ yielded a chloroplast location. However, the TargetP version 1.1 program predicted the protein to be in the secretion pathway with the presence of a signal peptide (not a chloroplast transit peptide). In addition, due to the presence of a signal peptide at the N-terminal of Fh3GT1, it was also analyzed for the presence of glycosylated residues using the NetNGlyc 1.0 server^2^. The prediction revealed that two putative glycosylation sites were found in two Asn residues: Asn-244 (NPTL) and Asn-386 (NGTM). Finally, in order to examine the subcellular distribution of Fh3GT1, transgenic *Arabidopsis* plants expressing this enzyme fused to the C terminus of GFP were produced, and the results showed that this protein seemed to be localized in both the nuclei and the cytosol (**Supplementary Figure S1**).

### Anthocyanin and Flavonol Analysis in Developing Flower

**FIGURE 2:**
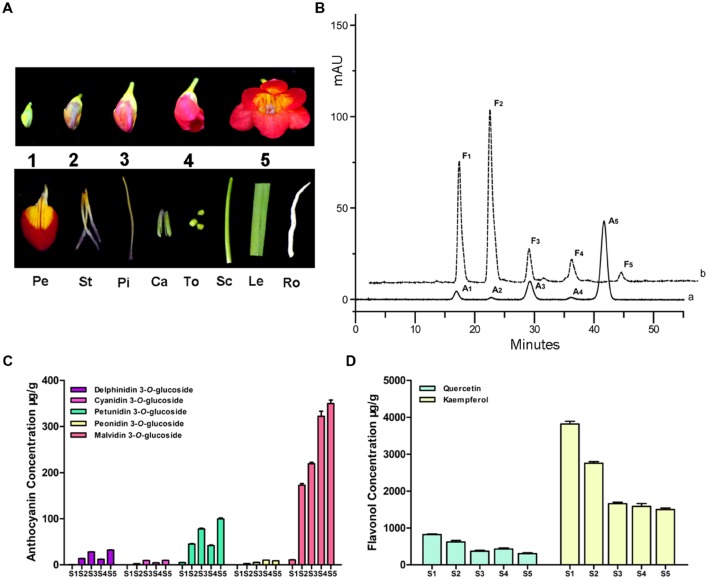
**Anthocyanin and flavonol component analysis in the flower of *F. hybrida*.** **(A)** The phenotypes of different samples. S1–S5, represent the flowers of different developmental stages; Pe, petals; St, stamens; Pi, pistils; Ca, calyxes; To, toruses; Sc, scapes; Le, leaves; Ro, roots. **(B)** High performance liquid chromatography (HPLC) profiles of anthocyanins and flavonols in flowers. (a) monitored at 520 nm for anthocyan in (b) monitored at 360 nm for flavonol. **(C)** Quantitative analysis of individual anthocyanin during flower development. **(D)** Quantitative analysis of individual flavonol during flower development. Data represent means ± SD of three biological replicates.

For determining the possible correlation between the presence of specific flavonoid glycoside derivatives and *Fh3GT1* expression levels, anthocyanins and flavonols were identified and quantified at five flower developmental stages (S1–S5) (**Figure 2A**). For anthocyanin, a total of five peaks (A_1_–A_5_) were identified in flowers (**Figure 2B**). Based on the MS and comparison to authentic standards, these peaks were identified as delphinidin 3-*O*-glucoside, cyanidin 3-*O*-glucoside, petunidin 3-*O*-glucoside, peonidin 3-*O*-glucoside, and malvidin 3-*O*-glucoside, respectively (**Supplementary Table S2**). Among these anthocyanins, malvidin 3-*O*-glucoside was the most abundant anthocyanin detected at all times (accounting for 64.53–82.48% of the total content), followed by petunidin 3-*O*-glucoside, delphinidin 3-*O*-glucoside, cyanidin 3-*O*-glucoside and peonidin 3-*O*-glucoside (**Figure 2C**). But other kinds of basic anthocyanin derivatives, pelargonidin glycosides, were not detected. Furthermore, we also assayed flavonols (F_1_–F_5_), which were most abundant in S1 stage. Of these flavonols, kaempferol glycosides were predominant, with minor quantities of quercetin glycosides observed (**Figure 2D**). However, myricetin glycosides were undetectable throughout the flower development. In accordance with the flower deep coloration, the total anthocyanins levels peaked dramatically (0.54 mg g^-1^ fresh weight, **Supplementary Figure S2A**) in S5 stage. On the contrary, total flavonols concentrations continued to decline gradually from S1 to a minimum at S5 (1.8 mg g^-1^ fresh weight, **Supplementary Figure S2B**).

### Expression Profile of *Fh3GT1* in *Freesia hybrida*

**FIGURE 3:**
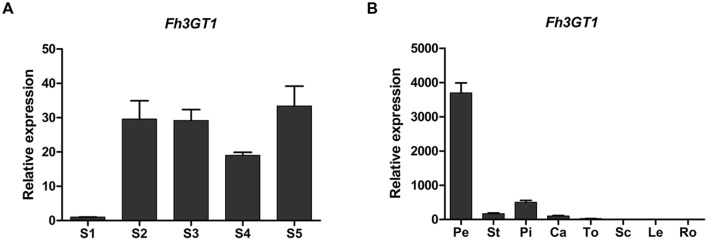
**Expression profiles of *Fh3GT1* gene in *Freesia hybrida*.** **(A)** Expression levels of *Fh3GT1* in flowers at different developmental stages. **(B)** Expression profiles of *Fh3GT1* in different tissues. Data represent means ± SD of three biological replicates.

To examine whether the expression pattern of *Fh3GT1* coincided spatially and temporally with anthocyanin and/or flavonol biosynthesis in *F. hybrida*, real-time PCR was performed to investigate its expression levels in flowers at five flower developmental stages and in different tissues. In flowers, *Fh3GT1* was detected in all developmental stages and reached the highest level at stage 5 (**Figure 3A**). Transcript analysis in different tissues revealed that *Fh3GT1* transcript was detected in all organs examined, and significantly higher levels of expression were observed in petals, pistils, and stamens. Comparatively, lower levels were seen in calyxes, toruses, scapes, leaves, and roots (**Figure 3B**) in which the anthocyanins were barely detected (data not shown). Taken together, the integrative expression pattern in combination with the accumulation of anthocyanins and flavonols suggest that *Fh3GT1* may participate in both the anthocyanin and the flavonol glycoside biosynthesis in *F. hybrida*.

### Biochemical Characterization of Fh3GT1

**FIGURE 4:**
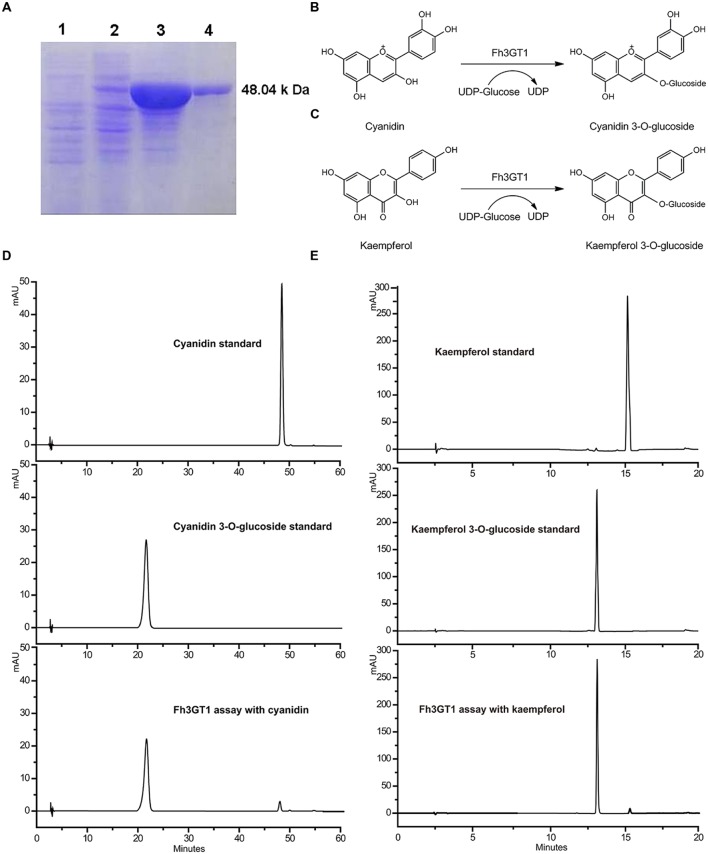
**Purification of recombinant Fh3GT1 and identification of reaction products.** **(A)** Expression of Fh3GT1 in *E. coli*. (1) total soluble protein from *E. coli* expressing pET-28a (+) vector (2) total soluble protein from *E. coli* expressing Fh3GT1 prior to induction with IPTG (3) 50 h after induction (4) purified Fh3GT1. **(B,C)** Reaction scheme of the enzymatic synthesis of cyanidin/kaempferol 3-*O*-glucoside from cyanidin/kaempferol and UDP-glucose. **(D,E)** HPLC analysis of the activities of Fh3GT1 toward cyanidin and kaempferol.

The full-length ORF of *Fh3GT1* was cloned into the pET-28a (+) expression vectors with N-terminal His tags and introduced into BL-21 (DE3) *Escherichia coli* cells. A large amount of recombinant protein accumulated in inclusion bodies. However, only minor proportion of recombinant protein was detected in the soluble fraction by Western-blot analysis with an anti-His-tag antibody. Then the methods were optimized till sufficient soluble protein was obtained. Coomassie Blue -stained SDS-PAGE of the purified enzyme showed one major band of the expected size that matched well with the calculated molecular mass of Fh3GT1 (**Figure 4A**). Initial enzyme assays using UDP-glucose as the sugar donor demonstrated that the recombinant Fh3GT1 could catalyze the transfer of glucose to the 3-position of the anthocyanidin cyanidin (**Figures 4B,D**) and the flavonol kaempferol (**Figures 4C,E**). Correspondingly, recombinant protein expressing the empty vector did not glucosylate either of the substrates. The reaction products were monitored by HPLC-diode array detection (HPLC-DAD) and compared with the authentic standards. Subsequently, optimal reaction conditions for Fh3GT1 were determined using cyanidin and UDP-glucose. The recombinant enzyme showed maximum activity at 30°C and pH 8.0 which has been used in the analysis of the majority of plant UFGTs (Ford et al., 1998; Masayuki et al., 2000; Kim et al., 2006).

Substrate specificity studies for Fh3GT1 with a range of substrates were tested, including several anthocyanidins and flavonols whose glycosylated products had been detected in *F. hybrida*. In the presence of UDP-glucose, the recombinant Fh3GT1 was able to glucosylate all tested anthocyanidin and flavonol aglycones at their 3-position. As shown in **Table 1**, quercetin and delphinidin exhibited the highest rates of flavonol and anthocyanidin glucosylation, respectively. Pelargonidin and petunidin served as substrate to a similar extent, whereas malvidin showed the lowest glucosylation rates. With regard to sugar donor preference, both UDP-glucose and UDP-galactose were accepted by Fh3GT1. But UDP-galactose was transferred to the 3-position of delphinidin and cyanidin with a lower level of galactosyl transfer activity, as determined by HPLC-DAD and compared to authentic standards (delphinidin 3-*O*-galactoside and cyanidin 3-*O*-galactoside). Moreover, the recombinant Fh3GT1 enzyme was unable to further glucosylate anthocyanidin 3-*O*-glucoside (data not shown), as the primary anthocyanins of *F. hybrida* in flowers are anthocyanidin 3-*O*-glucosides (**Supplementary Table S2**). These results revealed that Fh3GT1 worked well on anthocyanidins and flavonols *in vitro* preferentially as a glucosyltransferase with strict regiospecificity for the 3-position of anthocyanins and flavonols.

**Table 1:** Relative activity of Fh3GT1 toward several substrates.

Substrate	Sugar donor	Product	Relative activity (%)
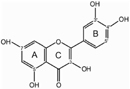	UDP-Glu	Quercetin 3-*O*-glucoside	100^a^
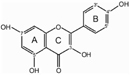	UDP-Glu	Kaempferol 3-*O*-glucoside	42.30
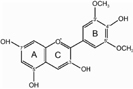	UDP-Glu	Malvidin 3-*O*-glucoside	8.29
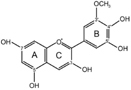	UDP-Glu	Petunidin 3-*O*-glucoside	13.10
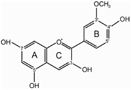	UDP-Glu	Peonidin 3-*O*-glucoside	32.33
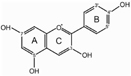	UDP-Glu	Pelargonidin 3-*O*-glucoside	14.93
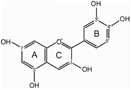	UDP-Glu	Cyanidin 3-*O*-glucoside	28.94
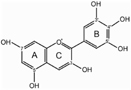	UDP-Glu	Delphinidin 3-*O*-glucoside	33.25
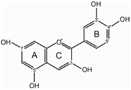	UDP-Gal	Cyanidin 3-*O*-galactoside	19.23
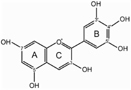	UDP-Gal	Delphinidin 3-*O*-galactoside	29.64


*^a^ The relative activity was calculated by the activity toward quercetin as 100%.*

*100 μM substrate and 10 mM sugar were used and the reaction was incubated at 30°C for 5 min.*

*Activity values are the mean of three independent determinations.*

The kinetic parameters of Fh3GT1 were examined using the best substrates of anthocyanidin and flavonol as determined by *in vitro* substrate specificity studies. By fixing the concentration of UDP-glucose and varying the concentration of substrates, hyperbolic saturation curves were obtained. Then the apparent *K*_m_ values were calculated based on the Lineweaver-Burk plot and listed in **Table 2**. According to the results, recombinant Fh3GT1 enzyme used quercetin most efficiently, although a relatively high affinity toward both delphinidin and peonidin was observed.

**Table 2:** Kinetic parameters for the recombinant Fh3GT1 protein.

Substrate	K_m_ (μM)	k_cat_ (s^-1^)	k_cat_/K_m_ (s^-1^M^-1^)
Quercetin	98.47 ± 4.96	4.59 ± 0.035	48.51 × 10^3^
Delphinidin	58.88 ± 1.68	0.53 ± 0.010	9.06 × 10^3^
Peonidin	56.08 ± 2.54	0.50 ± 0.011	8.97 × 10^3^


*Data are the means ± SD of three independent experiments.*

**FIGURE 5:**
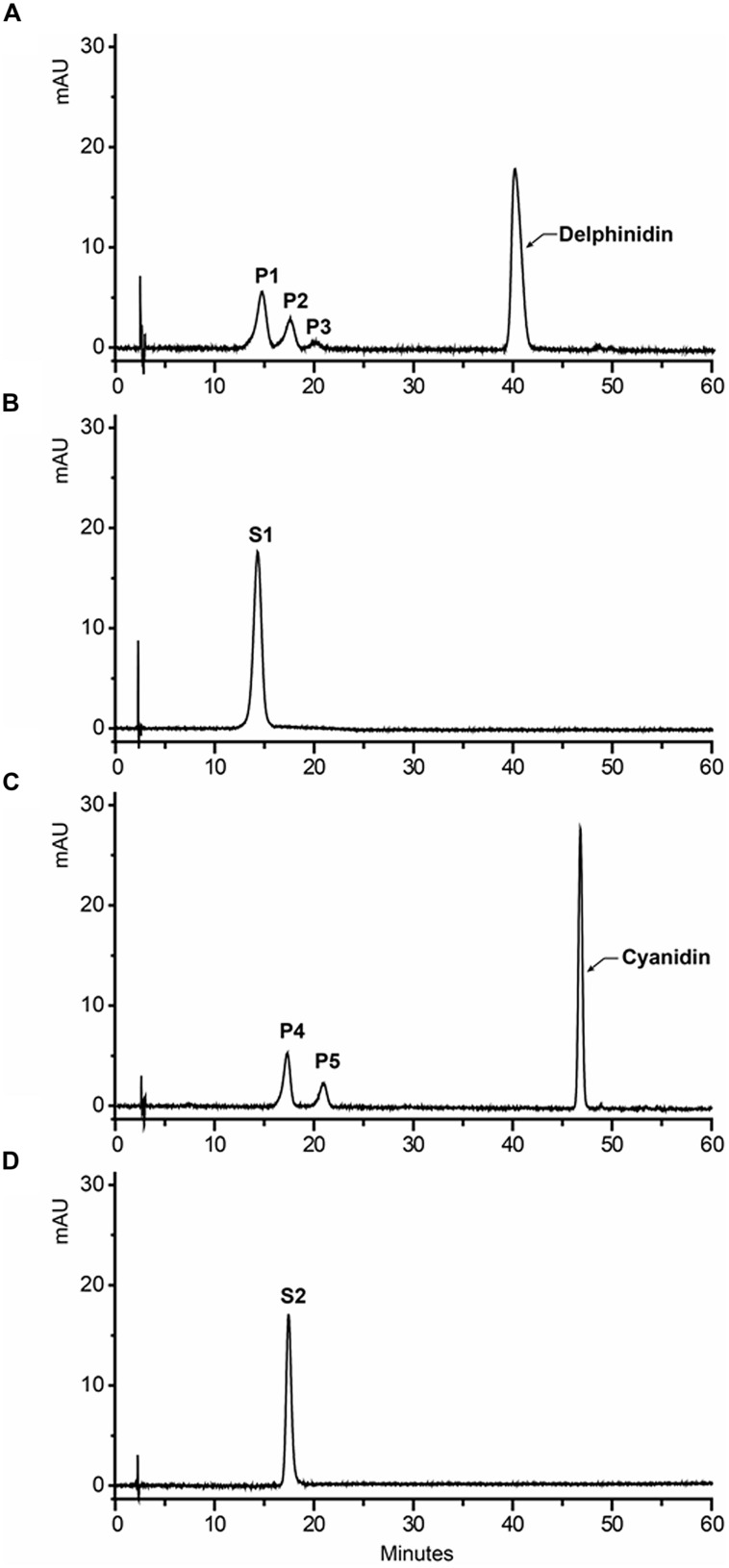
**HPLC profiles of Fh3GT1 reaction products with delphinidin/cyanidin and UDP-galactose.** **(A)** Delphinidin reaction products. **(B)** Authentic delphinidin 3-*O*-galactoside. **(C)** Cyanidin reaction products. **(D)** Authentic cyanidin 3-*O*-galactoside.

It has been suggested that concentration of the sugar donor could affect the regiospecificity of glycosyltransferase. Thus, 1 mM UDP-glucose and UDP-galactose were used as sugar donors with delphinidin and cyanidin as substrates to investigate the regiospecificity of Fh3GT1. In the case of UDP-glucose, one major reaction product was produced: the product was determined to be delphinidin and cyanidin 3-*O*-glucoside, while the amounts of other products were too low to be characterized (data not shown). However, HPLC analysis of the reaction products of UDP-galactose showed at least two peaks were different from substrates. HPLC-ESI-MS analyses of all reaction products were performed and the results indicated that molecular weight of each product increased 162 Da when compared to the weight of substrates. These results demonstrated that only one molecule glucose/galactose had been transferred to the substrates. In addition, the regiospecificity of Fh3GT1 could also be determined by observing the hypsochromic shift of UV spectra between Fh3GT1 reaction products and substrates. It has been reported that glycoslation at either C-3 or C-4′ hydroxyl group produce a hypsochromic shift, whereas glycoslation at the C-7 hydroxyl group showed no effect (Thomas et al., 1997; Kramer et al., 2003). The reaction products of delphinidin showed three peaks (P1-P3 in **Figure 5A**). The retention time of P1 was indistinguishable from that of the authentic delphinidin 3-*O*-galactoside (**Figure 5B**), revealing that P1 was 3-*O*-galactoside of delphinidin. P2 exhibited a hypsochromic shift, but P3 did not (**Table 3**). This suggested that P2 and P3 were likely to be delphinidin 4′-*O*-galactoside and delphinidin 7-*O*-galactoside, respectively. Fh3GT1 yielded two reaction products with cyanidin (P4–P5 in **Figure 5C**), P4 was determined to be cyanidin 3-*O*-galactoside by comparison with the authentic standard (**Figure 5D**) and P5 was likely cyanidin 4′-*O*-galactoside according to hypsochromic shift (**Table 3**).

**Table 3:** Structure assignment of Fh3GT1 reaction products.

Compounds	Retention time (min)	UV (nm)	Structure assignment
Delphinidin	40.12	254,531	Delphinidin aglycon
S1	14.74	251,521	Delphinidin 3-*O*-galactoside
P1^a^	14.65	250,520	Delphinidin 3-*O*-galactoside
P2^b^	17.43	251,520	Delphinidin 4′-*O*-galactoside
P3^b^	19.92	251,533	Delphinidin 7-*O*-galactoside
Cyanidin	46.73	255,526	Cyanidin aglycon
S2	17.44	254,515	Cyanidin 3-*O*-galactoside
P4^a^	17.17	255,515	Cyanidin 3-*O*-galactoside
P5^b^	20.84	255,516	Cyanidin 4′-*O*-galactoside


*^a^The reaction products were assigned according to comparison of HPLC retention time and UV spectra with authentic compounds.*

*^b^The reaction products were assigned according to the hypsochromic shift.*

### Fh3GT1 Functions as A 3-*O*-glucosyltransferase in Anthocyanin and Flavonol Biosynthesis *In Vivo*

**FIGURE 6:**
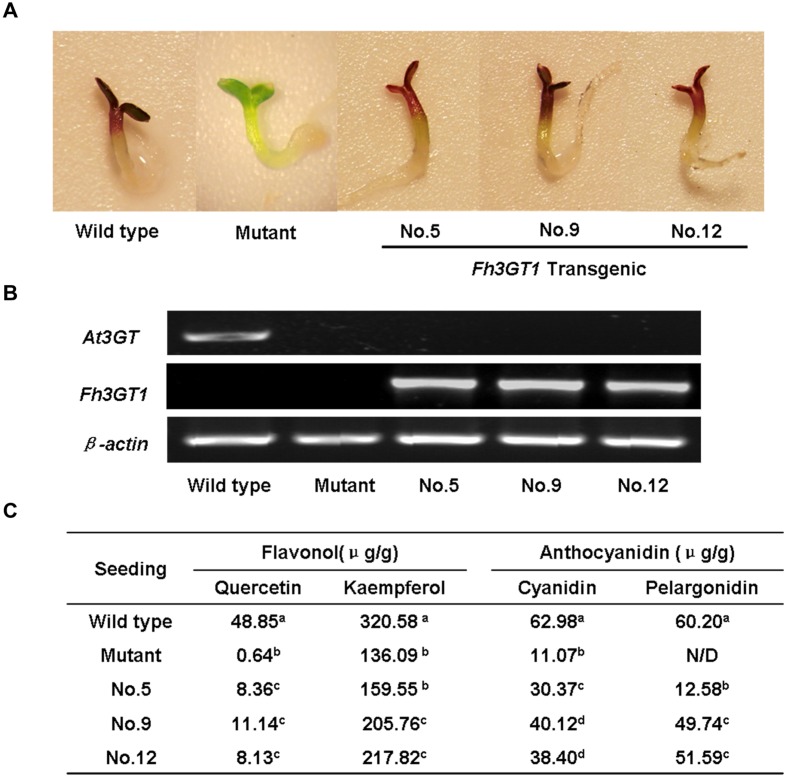
**Complementation of the pigmentation of *Arabidopsis* mutant seedlings with *Fh3GT1* gene.** **(A)** Phenotypes of wild-type, mutant (*UGT78D2*) and transgenic *Arabidopsis* seedlings. **(B)** Expressional analysis of the *At3GT* and *Fh3GT1* gene by reverse transcription polymerase chain reaction in wild-type, mutant and transgenic lines. **(C)** Contents of anthocyanins and flavonols in *Arabidopsis* seedlings. Data correspond to means of three biological replicates. Means with different letters within the same column are significantly different at the 0.01 level of probability. N/D, not detected.

To further confirm that Fh3GT1 has 3GT activity *in vivo*, the cDNA under the control of 35S promoter was transferred into the *Arabidopsis* mutant (*UGT78D2*) with reduced anthocyanidin and flavonol 3-*O-*glucosyltransferase activity. After selecting on 1/2 MS medium with kanamycin, more than twelve independent transgenic *Arabidopsis* lines were produced, and three of them (NO.5, NO.9, NO.12) were chosen for further analysis. Seeds of the wild-type, *Arabidopsis* mutant, and T2 transgenic lines were germinated and grown on anthocyanin gene induction media. Seedlings of wild-type and transgenic plants expressing *Fh3GT1* had anthocyanin pigmentation in the cotyledons and hypocotyls (**Figure 6A**), whereas the mutant transformed with the empty vector were green (not shown). Presence of the *Fh3GT1* and *Arabidopsis 3GT* (*At3GT*) was verified by RT-PCR using primers PF1/PR1 and *At3GT*F/*At3GT*R. PCR results showed that all transgenic lines expressed *Fh3GT1* and lacked the transcript of *At3GT* (**Figure 6B**).

**FIGURE 7:**
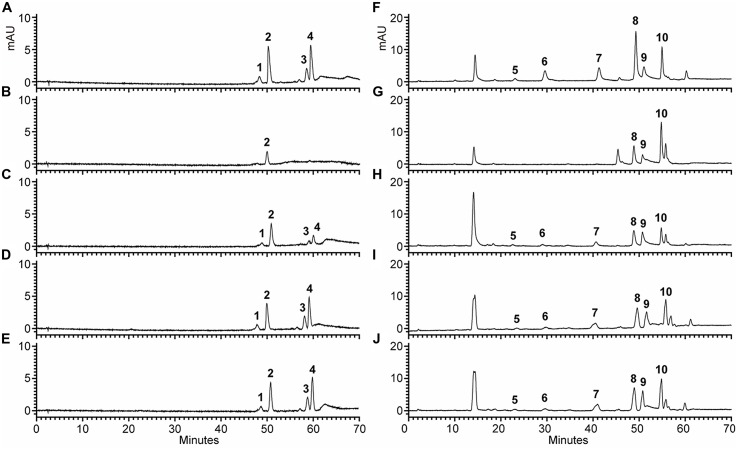
**High performance liquid chromatography analyses of anthocyanins and flavonols in transgenic *Arabidopsis* seedlings.** **(A–J)**. HPLC chromatograms of the samples from seedlings of wild type, mutant and transgenic lines. **(A,F)** wild type, **(B,G)** Mutant, **(C,H)** NO.5, **(D,I)** NO.9, **(E,J)** NO.12. **(A–E)** Absorbance at 520 nm for analysis of anthocyanins. **(F–J)** Absorbance at 360 nm for analysis of flavonols.

To examine the change of anthocyanin and flavonol more clearly in transgenic lines, T2 seedlings from each line cultured on anthocyanin induction media were extracted with extraction solvent mixture and analyzed by HPLC, and the MS data of these peaks were obtained and summarized in **Supplementary Table S3**. As shown in **Figure 7**, the *Arabidopsis* mutant had reduced peak area for some peaks of anthocyanin (monitored at 520) and flavonol (monitored at 360) relative to the wild type control. As expected, the pooled seedlings carrying *Fh3GT1* cDNA could restore these peaks, though lower levels of anthocyanin and flavonol were detected in transgenic plants (**Figure 6C**). Overall, our results strongly suggested that *Fh3GT1* had similar activity to *At3GT*, a 3GT gene participated in anthocyanin and flavonol biosynthesis *in vivo*.

## Discussion

Sequence analysis revealed that *Fh3GT1* was most closely related to anthocyanidin *3GT* from *Iris hollandica* (*IhAn3GT*) and they were clustered into the same clade of the phylogenetic tree (Sui et al., 2011). However, these two enzymes differ considerably in their substrate specificities. For example, Fh3GT1 showed highest activity toward quercetin, whereas IhAn3GT used malvidin as its best substrate (Yoshihara et al., 2005). The lack of congruence between substrate specificities and phylogenetic position has been observed in other UFGTs like VLOGT2 from grape (*Vitis labrusca*) and two UFGTs from onion (Kramer et al., 2003; Hall et al., 2011). These results are in agreement with the past suggestions that function and specificity of UFGT can not predict based on the primary sequence alone (Dhaubhadel et al., 2008). Coupling the tissue-specific expression with metabolites analysis has been known as an efficient method to confirm the glycosyltransferase functions. In this study, metabolite analysis and molecular approaches were used to investigate Fh3GT1-metabolite correlations for determining the role of Fh3GT1 in Freesia. Furthermore, analysis of substrate specificity *in vitro* and the use of transgenic *Arabidopsis* lines expressing *Fh3GT1* provided strong evidence demonstrating the biochemical function of Fh3GT1.

Based on the metabolites found in the flowers, the anthocyanin biosynthetic pathway in Freesia was proposed and presented in **Figure 1**, all of the six basic anthocyanin aglycons could be synthesized except pelargonindin derivatives. In previous studies, several plant species, such as *Petunia hybrida* and *Cymbidium hybrida*, have been found unable to produce pelargonidin derivatives because of the DFR substrate specificities. In these two plants, DFRs showed almost undetectable catalytic activities against dihydrokaempferol (DHK) (Joseph et al., 1998; Johnson et al., 1999), and we also found that the DFRs isolated from the Freesia flowers could not utilize DHK as substrates (unpublished date). On the other hand, DHK might be converted to dihydromyricetin (DHM) by F3′5′H before reduced by DFR or it fails to compete substrates with FLS.

*Freesia hybrida* accumulates both anthocyanins and flavonols, while anthocyanins accumulate to visible levels only in flower tissues. During the flower development, the level of anthocyanin gradually increased and peaked at stage 5, whereas flavonols reached its highest level at stage 1 (**Supplementary Figure S2**). Transcripts of *Fh3GT1* were detected in all examined samples, not just the sites of visible anthocyanin accumulations. This expression pattern is similar to that of *ANL1* in *Arabidopsis* (Kubo et al., 2007) and may suggest that *Fh3GT1* has a role in biosynthesis of anthocyanin and flavonol glycoside *in vivo*. Moreover, *Fh3GT1* was the most highly expressed at stage 5 (**Figure 3A**) and in petals (**Figure 3B**), respectively, and this expression profile showed the strongest correlation with the presence of anthocyanins rather than flavonols. It is therefore likely that *Fh3GT1* prefers to glycosylate the endogenous anthocyanidin in *F. hybrida.* Similar to *3GT* from black soybean (Kovinich et al., 2010), transfer of *Fh3GT1* into the *Arabidopsis* mutant also restored the biosynthesis of both anthocyanin and flavonol glycoside, confirming the functionality of Fh3GT1 as a 3GT *in vivo*. As shown in **Figure 6C**, anthocyanin levels were restored to a more degree (20.89–85%) than flavonols (7.31–25.49%) in transgenic *Arabidopsis* compared to the wild type control. These results further demonstrate that Fh3GT1 prefers anthocyanidin as substrates *in vivo*.

To date, all recombinant 3GTs characterized *in vitro* display unique substrate specificities. Some prefer anthocyanidins (Ford et al., 1998; Jun et al., 2004; Yoshihara et al., 2005; Almeida et al., 2007), while others prefer flavonols or other flavonoids (Modolo et al., 2007; Owens and McIntosh, 2009). On the basis of our *in vitro* biochemical assays, Fh3GT1 recombinant enzyme recognizes both anthocyanidins and flavonols as substrates, and has a clear preference for flavonols (**Table 1**). However, these results are inconsistent with those presented above demonstrating Fh3GT1 prefers to catalyze the glucosylation of anthocyanidin *in vivo*. Similar arguments have been proposed in UGT78K1, a 3GT of *Medicago truncatula* showed relatively low activity to cyanidin *in vitro* when compared to other flavonoid substrates (Modolo et al., 2007), but was demonstrated to play a critical role in the biosynthesis of anthocyanin *in vivo* (Peel et al., 2009). These results indicate that the enzymes involved in natural product biosynthesis are promiscuous, and the relative concentrations of potential substrates might be one of the most critical factors to determine their *in vivo* activity. Additionally, the recombinant Fh3GT1 could catalyze the transfer of a glycosyl moiety from sugar donor to the 3-position of pelargonidin *in vitro*, but no pelargonidin 3-*O*-glucoside was detected in *F. hybrida.* This suggests that the substrate recognition of Fh3GT1 is broader *in vitro*, and the branch of pelargonindin biosynthesis is blocked before Fh3GT1.

Fh3GT1 utilizes both UDP-glucose and UDP-galactose as sugar donors. The preference of UDP-glucose as the sugar donor is consistent with recent demonstration that the last residue of the PSPG box is a glutamine (Kubo et al., 2004). Meanwhile, the utilization of UDP-galactose as sugar donor also suggests that sugar donor specificity between glucose and galactose is probably determined not only by the last residue but also by other residue(s) in the PSPG box (Montefiori et al., 2011). Apparent *K*_m_ values for characterized 3GTs from various species vary greatly with values ranging from 1.5 to 400 μM (Saleh et al., 1976; Veljanovski and Constabel, 2013). The *K*_m_ of Fh3GT1 is within this range, and the relatively low *K*_m_ value for peonidin suggests it is likely to be the natural substrate for this enzyme *in vivo*. However, quantitative analyses of anthocyanidin glycosides in *F. hybrida* revealed that malvidin 3-*O*-glucoside was the most abundant anthocyanin (**Figure 2C**). This implies that *F. hybrida* has another type of 3GT recognizing malvidin as its natural substrate, because UFGTs in the flavonoid pathway are usually encoded by a multi-gene family in many plant species (Caputi et al., 2012).

Enzymatic studies of recombinant protein revealed some unique properties of Fh3GT1 toward its substrate. It has been reported that many glycosyltransferases involved in the flavonoid biosynthesis display high regiospecificity to a specific position of the hydroxy group but low substrate specificity to the flavonoid structure (Hansen et al., 2003). For instance, 3GTs of gentian, grape and petunia can glycosylate the 3-position of cyanidin, pelargonidin, and delphinidin (Yoshikazu et al., 1996; Ford et al., 1998; Mami et al., 2002). The gentian 5GT catalyzed the glycosylation of 5-hydroxy group of several anthocyanidin 3-glycosides (Nakatsuka et al., 2008). However, the regiospecificity of Fh3GT1 is determined by both the concentration and the kind of sugar donor. In contrast to the data of 1 mM sugar dornor, only anthocyanidin 3-*O*-glycoside was detected when 10 mM sugar dornor was used (**Table 1**). Experiments with recombinant Fh3GT1 showed galactosylation of the 3-OH, 4′-OH, and 7-OH with a preference for the 3-OH (**Table 3**). While in the case of glucose, Fh3GT1 glucosylated predominantly in the 3-position, and other products were produced only in trace amounts. This result is congruent with the fact that Freesia contains mainly anthocynidin 3-*O*-glucoside which is glucosylated in the 3-position. Further analyses suggest that 7-OH galactosylation is governed by the presence or absence of a hydroxyl group at C-5′. When this group is present (delphinidin), 7-OH galactosylation is observed, when absence (cyanidin) 7-OH galactosylation is not detected. Further investigation should be performed to confirm whether the presence of 5′-OH is a determinant for 7-OH galactosylation.

## Conclusion

The enzyme reported in this study can be identified as flavonoid 3-*O*-glycosyltransferase with UDP-glucose as the preferred sugar donor. Biochemical analysis together with the *in planta* data reveal its involvement in biosynthesis of flavonoid glucosides in *F. hybrida.* Moreover, this enzyme exhibits broad range substrate specificity toward flavonoids, including substrate (pelargonidin) that does not naturally occur in Freesia. This suggests the substrate versatility of Fh3GT1 could be even broader than described here. Interestingly, Fh3GT1 was also found to preferentially transfer a galactose group to the 3-OH, 4′-OH, and 7-OH of flavonoids. Due to the wide range substrate specificity and low regiospecificity of Fh3GT1, it could be an attractive enzyme to engineer the flavonoid diversity.

## Author Contributions

WS performed the research, wrote, and revised this manuscript; LL, XM, YL, FG, SW, and XL performed the experiments and helped analyze data. XG and LW designed the experiments, discussed results, and revised this manuscript. All authors have participated in this research and approved the final manuscript.

## Conflict of Interest Statement

The authors declare that the research was conducted in the absence of any commercial or financial relationships that could be construed as a potential conflict of interest.

## Footnotes

1 http://psort.nibb.ac.jp

2 http://www.cbs.dtu.dk/services/NetNGlyc/

## Abbreviations

3GT: Flavonoid 3-*O*-glucosyltransferase
5GT: Anthocyanin 5-*O*-glucosyltransferase
ANS: Anthocyanidin synthase
CHI: Chalcone isomerase
CHS: Chalcone synthase
DFR: Dihydroflavonol-4-reductase
F3′5′H: Flavonoid 3′ 5′-hydroxylase
FLS: Flavonol synthase
HPLC: High performance liquid chromatography
UFGT: UDP flavonoid glycosyltransferases
