# Supplementary material for: Biochemical and Molecular Characterization of a Flavonoid 3-O-glycosyltransferase Responsible for Anthocyanins and Flavonols Biosynthesis in Freesia hybrida
Source: Front Plant Sci. 2016 Mar 31;7:410. doi: 10.3389/fpls.2016.00410 (PMC4815329; doi:10.3389/fpls.2016.00410)

Supplementary Material:

Biochemical and molecular characterization of a flavonoid 3-*O*-glycosyltransferase responsible for anthocyanins and flavonols biosynthesis in *Freesia hybrida*

Wei Sun[1, 3], Lingjie Liang[1], Xiangyu Meng[1], Yueqing Li[1], Fengzhan Gao[1], Xingxue Liu[1], Shucai Wang[2], Xiang Gao[1, 2*], Li Wang[1*]

*Correspondence: Institute of Genetics and Cytology, Northeast Normal University, Changchun, 130024 China

Corresponding author: Xiang Gao, Li Wang

E-mail address: gaoxiang424@163.com (XG); wanglee57@163.com (LW)

SUPPLEMENTARY TABLES AND FIGURES

Supplementary Table S1. Primer sequences used in this study

Primer	Sequences 5'-3'
GFPF1	CGGTCTAGAATGGTGAGCAAGGGCGAG
GFPR1	CGGTCTAGACTTGTACAGCTCGTCTAC
GFPF2	CGGTCTAGAATGGTGAGCAAGGGCGAG
GFPR2	CGGGGATCCTTACTTGTACAGCTCGTC
PF1	TCCTCTGGTTCGTTGGGGACATC
PR1	GTTCCTCAGCATGTCGGTGTAGAG
18SF1	TCCTGATACGGGGAGGTAGTGACA
18SR1	ACTTGCCCTCCAATGGATCCTCG
PF2	CTCGAATTCCAGCAAGCAATGGGATCG
PR2	CGGAAGCTTCAGACTTCAGTCATATTCCGA
PF3	CGGTCTAGACAGCAAGCAATGGGATCG
PR3	CGAGGATCCCAGACTTCAGTCATATTCCGA
At3GTF	TGGCAGTTCTCGCTTTTCCTTTC
At3GTR	ACTTTACTCGATTCCAACCCTTC

Supplementary Table S2. The flavonoid profiles in acidic MeOH-H2O extracts of the *Freesia hybrida*

Peak number	Identifacation/tentative identification	Retention time (min)	λmax (nm)	ESI-MS (m/z)
A1	Delphinidin 3-O-glucoside	17.24	275/524	303.1[Del+H]+<br>465.0[Del+Glc+H]+
A2	Cyanidin 3-O-glucoside	23.06	272/514	287.0[Cya+H]+<br>449.0[Cya+Glc+H]+
A3	Petunidin 3-O-glucoside	29.59	276/526	317.1[Pet+H]+<br>479.1[Pet +Glc+H]+
A4	Peonidin 3-O-glucoside	36.49	272/515	301.2[Peo+H]+<br>463.0[Peo+Glc+H]+
A5	Malvidin 3-O-glucoside	41.89	277/527	331.1[Mal+H]+<br>493.0[Mal+Glc+H]+
F1	Quercetin derivatives	15.18	254/334	303.1<br>479.1<br>987.3
F2	Kaempferol derivatives	20.38	268/328	287.1<br>463.1<br>971.4
F3	Quercetin glucuronide	26.96	257/316	303.1[Qr+H]$^{+}$<br>479.1[Qr+Glu+H]$^{+}$
F4	Kaempferol derivatives	34.07	268/315	287.0<br>463.0<br>955.5
F5	Kaempferol derivatives	42.30	255/324	287

Flavonoids were quantified by measuring peak area (anthocyanin; at 520 nm, flavonol; at 360 nm) using a standard curve of reference compounds. Del, delphinidin; Cya, cyanidin; Pet, petunidin; Peo, peonidin; Mal, malvidin; Qr, quercetin; Glc, glucose.

Supplementary Table S3. **HPLC-DAD and HPLC-ESI-MS analysis of flavonoid in acidic MeOH-H2O extracts of the wild-type *Arabidopsis* and Fh3GT1 over-expressing lines**

Peak number	Identifacation/tentative identification	Retention time (min)	λmax (nm)	ESI-MS (m/z)	References
1	Cyanidin 3-*O*-[2″-*O*-(xylosyl) 6″-*O*-(*p*-*O*-(glucosyl) *p*-coumaroyl) glucoside] 5-*O*-[6″″-*O*-(malonyl) glucoside]	48.78	266/531	$287.1[Cy+H]^+$ $1137.4[M+H]^+$	Takayuki Tohge, Yasutaka Nishiyama et al., 2005
2	Cyanidin 3-*O*-[2″-*O*-(6‴-*O*-(sinapoyl) xylosyl) 6″-*O*-(p-*O*-(glucosyl)-*p*-coumaroyl) glucoside] 5-*O*-(6″″-*O*-malonyl) glucoside	50.84	269/535	$287.0[Cy+H]^+$ $1343.2[M+H]^+$	Stephen J. Bloora, Sharon Abrahamsb., 2002
3	Pelargonidin derivatives	58.81	278/526	$271.2[Pg+H]^+$	
4	Pelargonidin derivatives	59.92	284/535	$271.3[Pg+H]^+$	
5	Kaempferol 7-*O*-rhamnopyranoside	23.07	264/355	$287.2[Km+H]^+$ $432.7[M+H]^+$	Jin-Ying Gou, Felipe F. Felippes et al., 2011
6	Quercetin 3-*O*-rhamnoside 7-*O*-rhamnoside	29.59	265/347	303.0[Qr+H]+ 449.0[Qr+Rha+H]+	Graham., 1998
7	Kaempferol derivatives	40.96	260/353	$287.3[Km+H]^+$	
8	Kaempferol 3-*O*-glucoside 7-*O*-rhamnoside.	48.85	262/349	$287.2[Km+H]^+$ $433.1[Km+ Rha+H]^+$	Takayuki Tohge, Yasutaka Nishiyama et al., 2005
10	Kaempferol 3-*O*-rhamnoside 7-*O*-rhamnoside	54.83	265/342	$286.9[Km+H]^+$ $433.0[Km+ Rha+H]^+$	Jin-Ying Gou, Felipe F. Felippes et al., 2011

According to the analysis of standard, we found that anthocyanin has absorbance peak at both 520nm and 360nm, but flavonol only has absorbance peak at 360nm. HPLC results revealed that the retention time of peak 9 is same to peak 2.Therefore, we deduce that peak 9, which is part of the peak 2, is the kind of anthocyanin, and it was not used for quantitative analysis of flavonol.

Supplementary Figure S1. Subcellular localization of Fh3GT1. A. GFP alone. B. Fh3GT1-GFP.

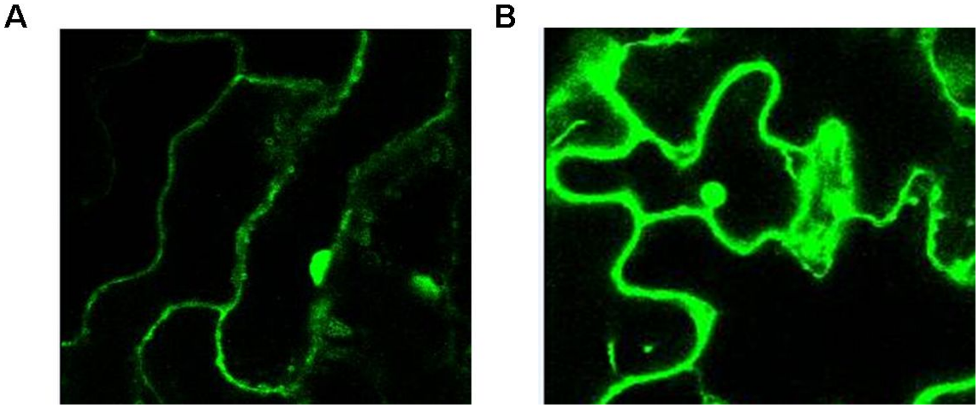

Supplementary Figure S2. Quantitative analyses of total anthocyanins and flavonols during flower development. A. Anthocyanins. B. Flavonols. Data represent means ± SD of three biological replicates.

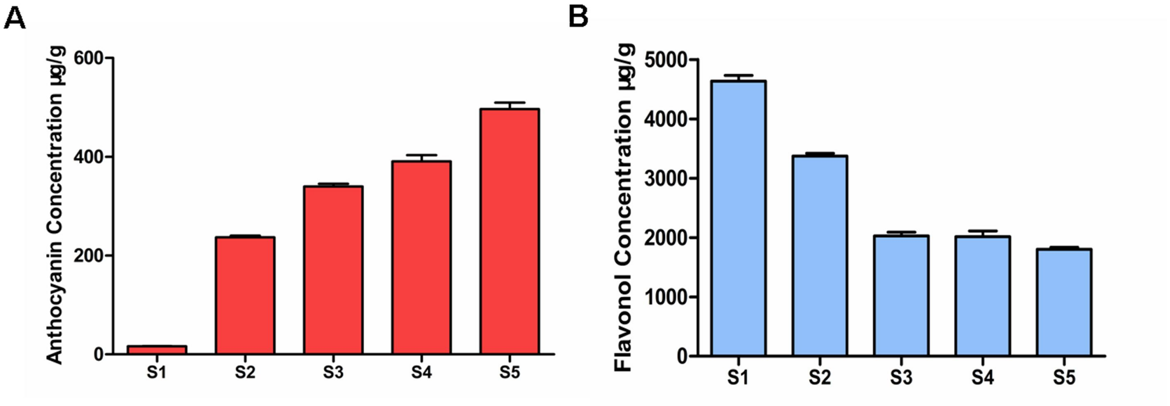

Supplement: Supplementary file 1 [file Data_Sheet_1.PDF]
